# Patient-centred access to health care: conceptualising access at the interface of health systems and populations

**DOI:** 10.1186/1475-9276-12-18

**Published:** 2013-03-11

**Authors:** Jean-Frederic Levesque, Mark F Harris, Grant Russell

**Affiliations:** 1 Institut national de santé publique du Québec, 190 Crémazie Est, Montréal, QC H2P1E2, Canada; 2 University of New South Wales, Sydney, NSW 2052, Australia; 3 Monash University, Wellington Road, Clayton, VIC 3800, Australia

**Keywords:** Access to healthcare, Accessibility, Utilisation of health services, Availability

## Abstract

**Background:**

Access is central to the performance of health care systems around the world. However, access to health care remains a complex notion as exemplified in the variety of interpretations of the concept across authors. The aim of this paper is to suggest a conceptualisation of access to health care describing broad dimensions and determinants that integrate demand and supply-side-factors and enabling the operationalisation of access to health care all along the process of obtaining care and benefiting from the services.

**Methods:**

A synthesis of the published literature on the conceptualisation of access has been performed. The most cited frameworks served as a basis to develop a revised conceptual framework.

**Results:**

Here, we view access as the opportunity to identify healthcare needs, to seek healthcare services, to reach, to obtain or use health care services, and to actually have a need for services fulfilled. We conceptualise five dimensions of accessibility: 1) Approachability; 2) Acceptability; 3) Availability and accommodation; 4) Affordability; 5) Appropriateness. In this framework, five corresponding abilities of populations interact with the dimensions of accessibility to generate access. Five corollary dimensions of abilities include: 1) Ability to perceive; 2) Ability to seek; 3) Ability to reach; 4) Ability to pay; and 5) Ability to engage.

**Conclusions:**

This paper explains the comprehensiveness and dynamic nature of this conceptualisation of access to care and identifies relevant determinants that can have an impact on access from a multilevel perspective where factors related to health systems, institutions, organisations and providers are considered with factors at the individual, household, community, and population levels.

## Background

Access to healthcare is central in the performance of health care systems around the world. In fact, the importance of service delivery for people has resulted in measurement of utilisation and access having a prominent role in the health policy literature [1,2]. However, access to health care remains a complex notion as exemplified by the varying interpretations of the concept across authors [3,4].

Etymologically, access is defined as a way of approaching, reaching or entering a place, as the right or opportunity to reach, use or visit [5]. Within health care, access is always defined as access to a service, a provider or an institution, thus defined as the opportunity or ease with which consumers or communities are able to use appropriate services in proportion to their needs [4,6].

Access has been conceptualised in numerous ways. While the term access is often used to describe factors or characteristics influencing the initial contact or use of services, opinions differ regarding aspects included within access and whether the emphasis should be put more on describing characteristics of the providers or the actual process of care [7]. Some authors view access more as an attribute of health services, noting the fact that services can be accessed or utilised by those requiring care [8]. While most authors do recognise the influence of characteristics of users as well as characteristics of providers on access, many put more emphasis on characteristics of health care resources that influence the utilisation of services, acting as a mediating factor between the ability to produce services and their consumption [9]. Penchansky is amongst those that more explicitly conceptualised access in terms of the fit between characteristics of providers and health services, and characteristics and expectations of clients [2]. Here, access may be conceived as the interface between potential users and health care resources, and would be influenced by characteristics of those who supply as well as those who utilise the services.

Access has often been defined as the use of health care, qualified by need for care [10]. It has also been defined as describing the costs incurred in receiving care, as the maximum attainable consumption, or as foregone utility [11].

Mooney sees access as a function of both supply and demand [12]. In this view, access to health care is a product of supply factors, such as the location, availability, cost and appropriateness of services, as well as demand factors, such as the burden of disease and knowledge, attitudes and skills and self-care practices [13-15].

This is in line with the notions of predisposing factors to utilisation on one side, and enabling and health system factors on the other [1]. Predisposing factors include an individual’s perception of an illness, as well as population-specific cultural, social, and epidemiological factors. Enabling factors include the means available to individuals for using health services. Health system factors comprise resources, structures, institutions, procedures, and regulations through which health services are delivered [1].

Frenk reserves the term access to denote the ability of the population to seek and obtain care. It thus refers to a characteristic of the population of potential or real users of services and is related to the concept of utilization power and resistance [7]. A theoretically attractive way to see access is to see it as the degree of adjustment between the characteristics of the population and those of the health care resources seeing access as a functional relationship between the population and medical facilities and resources, and which reflects the differential existence either of obstacles, impediments and difficulties or of factors that are facilitators for the beneficiaries of health care [7].

Although a conceptual vision of fit suggests that both resource and population characteristics can be modified to ensure continuing levels of access, only resources can be modified in the short-term [7]. In general, obstacles such as price of services, transportation time, and waiting time are more responsive to specific health policies than the broader social and economic characteristics of the population, such as income, transportation resources, or free time [7].

Andersen, conceptualising utilisation as realised access, has viewed utilisation (type, site, purpose, time interval) as determined by population characteristics (predisposing, enabling, need) and health systems’ characteristics (policy, resources, organization) [13,14]. In a similar manner, highlighting the relation between the concepts of utilisation and access, Donabedian highlighted the central role of characteristics of health resources with regards to facilitating or impeding the use of services by potential users [9]. Table 1 summarizes definitions and dimensions found in the literature.

**Table 1:** Definitions and dimensions of access to health care

**Authors**	**Definition**	**Dimensions**
Bashshur et al., 1971	Accessibility as the functional relationship between the population and medical facilities and resources, and which reflects the differential existence either of obstacles, impediments and difficulties, or of factors that are facilitators for the beneficiaries of health care	
Donabedian, 1973	Accessibility comprising the concept of degree of adjustment between resources and populations	
Salkever, 1976	Accessibility combining attributes of the resources and attributes of the population	Financial accessibility
		Physical accessibility
Aday & Andersen, 1974	Access as entry into the health care system	Predisposing factors
		Enabling factors
		Need for health care
Penchansky & Thomas, 1981		Affordability Accessibility
		Accommodation
		Availability
		Acceptability
Dutton, 1986	Utilisation viewed as the product of patients characteristics plus provider and system attributes	Financial
		Time
		Organizational factors
Frenk, 1992	Access as the ability of the population to seek and obtain care	
	Accessibility is the degree of adjustment between the characteristics of health care resources and those of the population within the process of seeking and obtaining care	
Margolis et al., 1995	The timely use of personal health services to achieve the best possible outcomes.	Financial
		Personal
		Structural
Haddad & Mohindra, 2002	The opportunity to consume health goods and services	Availability
		Affordability
		Acceptability
		Adequacy
Shengelia et al., 2003	Coverage: probability of receiving a necessary health intervention, conditional on health care need	Physical access
		Resource availability
	Utilization: quantity of health care services and procedures used	Cultural acceptability
		Financial affordability
		Quality of care
Peters et al. 2008	Access viewed as including actual use of services. A clear emphasis is given to consider both users and services characteristics in evaluation of access. The notion of fit between users and services is identified.	Quality
		Geographic accessibility
		Availability
		Financial accessibility
		Acceptability of services

The disaggregation of access into broad dimensions, such as geographical, economical or social aspects, permits more operational measures through the study of specific determinants of access to health care. However, measuring access is a complex task when trying to include dimensions other than merely availability of services. Access is often perceived as being predominantly an attribute of services and is determined by factors such as the availability, price and quality of health resources, goods and services. This perception could stem from the fact that it is factors amenable to policies and organisational aspects of care that should be targeted to improve access. Meanwhile, utilization, often used as a proxy of access (realised access is easier to measure than potential access) is influenced by the supply as well as the demand for services, including individual attributes such as preferences, tastes and information [12,16,17]. Others have added financial and physical barriers to utilisation as determinants of access to health care [8]. But access clearly goes further than an availability of health services. A more comprehensive view on access should consider factors pertaining to the structural features of the health care system (e.g. availability), features of individuals (consisting of predisposing and enabling factors) and process factors (which describe the ways in which access is realised) [4,18,19], and pertains to the dimensions of availability, accessibility, accommodation, affordability and acceptability [2]. Others have proposed dimensions related to factors such as geographic access, resource availability, cultural acceptability, financial affordability, and quality of care to health system coverage [1,20].

Despite ongoing preoccupation with access to health care, we consider that health services research and policy continues to be compromised by a lack of clarity of concepts of access and utilisation, lack of consensus on sub dimensions of access, and ongoing blurring of access as a concept and its determinants. The emergence of chronic disease and the increasing realisation that patients have a growing role in chronic care also highlight the need to revisit the concept of access to better incorporate patient-centred perspectives into population and system level approaches. The aim of this paper is to suggest a conceptualisation of access to health care describing broad dimensions and determinants that integrate demand and supply-side-factors and enabling the operationalisation of access to health care all along the process of obtaining care and benefiting from the services.

## Methods

This paper comes from a synthesis of published literature on the concept of access to healthcare. PubMed and CINAHL (Cumulative Index to Nursing and Allied Health Literature) databases were searched for papers using these keywords: access; accessibility, utilisation of health care services; availability; affordability; acceptability. These keywords were combined to restrict the number of articles retrieved. The words conceptualisation, concept and construct were appended to further narrow the search. Textbooks in the fields of public health and health administration were searched for chapters pertaining to access. Snowball retrieval of texts using the reference list of review papers and book chapters were used to complement the search. Finally, some manuscripts or documents listing publications related to the conceptualisation of access were retrieved on the web using readily available search engines (Google, Google scholar). Papers were screened and selected according to the presence of a conceptual proposal or discussion of the concept of access. Papers solely referring to a previously published manuscript were not retained for analyses and the authors referred to the original publications. The conceptualisation most often cited in papers pertaining to the conceptualisation of access to care and clearly identifying dimensions and determinants were retained to form the basis of the analysis and proposal of a framework. This synthesis was part of the PhD dissertation of the principal author.

## Results - revisiting the concept of access

### A definition of access as an opportunity

**Figure 1:**
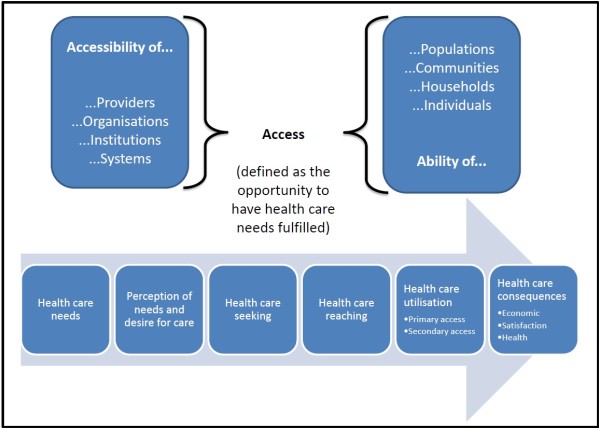
A definition of access to health care.

Here, access is defined as the opportunity to reach and obtain appropriate health care services in situations of perceived need for care [3,10,21,22]. Access is seen as resulting from the interface between the characteristics of persons, households, social and physical environments and the characteristics of health systems, organisations and providers [2]. Factors to consider could thus pertain to supply-side features of health systems and organizations, to demand-side features of populations, and to process factors describing the ways in which access is realised [4,23]. Therefore, we view access as the possibility to identify healthcare needs, to seek healthcare services, to reach the healthcare resources, to obtain or use health care services, and to actually be offered services appropriate to the needs for care (cf. Figure 1).

This opportunity is considered as different from the notion of accessibility, which describes the nature of the services that provide this opportunity. This framework positions the concept of utilisation as realised access [13]. Access thus in our view enables people to make the steps that enable them to enter in contact and obtain health care. As such, variations in access are conceptualised in terms of differences in the perception of needs for care, in healthcare seeking, in reaching and obtaining (or delay in obtaining), in the type and intensity of services received can potentially reveal variations in access. These different steps in the sequence that a patient will experience represent crucial transitions where barriers to access can be revealed.

Clearly such a broad scope of access all along the pathway of utilisation from perception of need until benefiting from care is not always adopted in the scientific literature. A narrow domain sees access as pertaining to a restricted part of the health care seeking process often starting from the search for care and continuing to the initiation of care. An intermediate domain adds the continuing care aspects in the evaluation (access does not pertain to the first-contact only but is relevant each time a person tries to access a source of care). In addition, access to some services may be contingent on use of other services (e.g. use of primary care providers or case managers in order to access specialist or allied health professional care). A broader domain includes the delay between the desire for care and the actual search for care (including aspects such as trust in and expectation towards the health care system, health literacy, knowledge about services and their usefulness). For some, under the broad domain, the study of access relates to similar aspects as the study of utilisation of health care services [7].

### Five dimensions of access capturing supply-side and demand-side determinants

**Figure 2:**
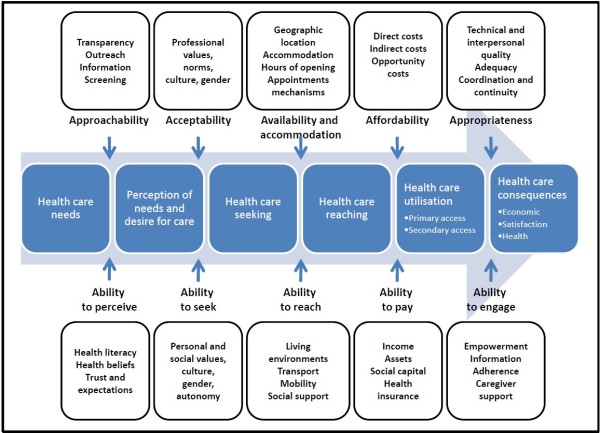
A conceptual framework of access to health care.

We conceptualise five dimensions of accessibility of services as represented in the upper part of Figure 2: 1) Approachability; 2) Acceptability; 3) Availability and accommodation; 4) Affordability; 5) Appropriateness. These five dimensions relate to various proposed dimensions [1-3,24]. Five corresponding abilities of persons interact with the dimensions of accessibility to generate access. As shown in the lower part of Figure 2, these dimensions of abilities include: 1) Ability to perceive; 2) Ability to seek; 3) Ability to reach; 4) Ability to pay; 5) and Ability to engage.

Approachability relates to the fact that people facing health needs can actually identify that some form of services exists, can be reached, and have an impact on the health of the individual. Services can make themselves more or less known among various social or geographical population groups. Various elements such as transparency, information regarding available treatments and services and outreach activities could contribute to make the services more or less approachable. Complementary to this notion of approachability of services, the notion of ability to perceive need for care among populations is crucial and determined by such factors such as health literacy, knowledge about health and beliefs related to health and sickness.

Acceptability relates to cultural and social factors determining the possibility for people to accept the aspects of the service (e.g. the sex or social group of providers, the beliefs associated to systems of medicine) and the judged appropriateness for the persons to seek care. For example, a society forbidding casual physical contact between unmarried men and women would reduce acceptability of care and acceptability to seek care for women if health service providers are mostly men. It may be that some services are inequitable in the way they are organized, making them unacceptable to some sections of the community that they are intended to serve [6]. Ability to seek health care relates to the concepts of personal autonomy and capacity to choose to seek care, knowledge about health care options and individual rights that would determine expressing the intention to obtain health care. A good example would be female discrimination regarding the initiation of care or abuse and neglect discouraging ethnic minorities to seek care. This relates to the challenge of ensuring that care meets the needs of different cultural, socioeconomically disadvantaged and vulnerable populations. Because different groups may judge appropriateness and quality differently, this is an important challenge [25].

Availability and accommodation refers to the fact that health services (either the physical space or those working in health care roles) can be reached both physically and in a timely manner. Availability constitutes the physical existence of health resources with sufficient capacity to produce services (existence of productive facilities) [7]. It results from characteristics of facilities (e.g. density, concentration, distribution, building accessibility), of urban contexts (e.g. decentralisation, urban spread, and transportation system) and of individuals (e.g. duration and flexibility of working hours). It also relates to characteristics of providers (e.g. presence of the health professional, qualification) and modes of provision of services (e.g. contact procedure and possibility of virtual consultations). Access is restricted if available resources are unevenly distributed around a country, or across levels of care (with specialty care developed at the expense of primary care) [6]. Ability to reach health care relates to the notion of personal mobility and availability of transportation, occupational flexibility, and knowledge about health services that would enable one person to physically reach service providers. Restricted mobility of the aged and handicapped, or the inability of casual workers to be absent from work to consult medical providers would be examples of these.

Affordability reflects the economic capacity for people to spend resources and time to use appropriate services. It results from direct prices of services and related expenses in addition to opportunity costs related to loss of income. Furthermore it can vary by type of services and depends on the capacity to generate the resources to pay for care (e.g. mode of payment, mobilisation of resources). Economic studies of utilization models demand using variables such as price of care, travel time and the opportunity costs linked to it, patient’s income, perceived quality of care, provider behaviour, etc. These models give useful information about elasticity of demand for different types of health services [1]. Ability to pay for health care is a widely used concept within the health services and health economics literature [8,26]. It describes the capacity to generate economic resources - through income, savings, borrowing or loans - to pay for health care services without catastrophic expenditure of resources required for basic necessities (e.g. sale of home). Poverty, social isolation, or indebtedness would be examples of factors restricting the capacity of people to pay for needed care.

Appropriateness denotes the fit between services and clients need, its timeliness, the amount of care spent in assessing health problems and determining the correct treatment and the technical and interpersonal quality of the services provided [7,27]. Adequacy relates to the appropriateness (what services are provided) and quality (the way in which they are provided) of health services and its integrated and continuous nature [7,27]. Clearly, the content and effectiveness of health services and goods one has the opportunity to utilise matters [1]. Opportunity to utilise only services of poor quality in this sense is seen as restriction of access to health care. Some suggest that these dimensions - acceptability and adequacy - should not be part of access [7]. Our reasoning is that one should not have access to health care based on geographical and organisational availability and affordability alone, but that access encompasses the possibility to choose acceptable and effective services. The opportunity for a person to utilise the services of untrained practitioners (e.g. witch doctors, healers) cannot be equated to the opportunity for another person - wealthier - to utilise highly specialised services, if these services generate different health outcomes or satisfaction towards services. Utilisation of services with inherently differential technical qualities - either through the utilisation of different types of providers or through differential prescription practices - cannot be seen as equally appropriate care. Finally, ability to engage in health care would relate to the participation and involvement of the client in decision-making and treatment decisions, which is in turn strongly determined by capacity and motivation to participate in care and commit to its completion. This dimension is strongly related to the capacity to communicate as well as notions of health literacy, self-efficacy and self-management in addition to the importance of receiving care that is actually appropriate for the person, given its resources and skills. Access to optimal care ultimately requires the person to be fully engaged in care and this is seen as interacting with the nature of the service actually offered and provided.

## Discussion

### A dynamic and cumulative perspective on access

The various dimensions of access identified are not completely independent constructs [3]. They often influence each other and act at different times during an episode of illness and care [28-30]. As an example, geographic availability can interact with affordability of transportation in influencing access to health services. These constructs should thus be considered as interrelated [3].

We view access to health care services as resulting from the interaction of determinants pertaining to characteristics of individuals (e.g. the place where they live, their economic resources and their social status) and of services (e.g. quantity, location of facilities, costs). It is not simply the cost of services themselves that determines if services are affordable but also the capacity of people to pay for these services. Similarly, the location of a health facility will have an impact on access to health care depending on the patterns of settlement of the population it serves and their capacity to travel to the health service. Defining access as an attribute of services emphasises the fact that health services should respond to the population’s characteristics to ensure the people’s capacity to use the services when facing need for care [6]. Characteristics of resources, individuals and communities can determine various dimensions simultaneously. Figure 2 illustrates some determinants of access pertaining to providers, health facilities, health systems (supply side), individuals, households (demand side), and urban areas (context).

This framework provides clues into what are the dimensions that relate to various abilities of patients that healthcare services characteristics interact with in providing access to care along the continuum of health care seeking. What makes it patient-centred is that this framework places at the centre of analysis the actual process of seeking care, including the various stages that a patient has to go through to actually receive the needed care. This framework could provide the basis for a stronger operational measurement of the various aspects related to the abilities of patients in interacting with health services. In addition, it may provide guidance into policies aiming at addressing certain gaps in patients’ abilities in order to promote access.

### A framework supported by previous work on the determinants of utilisation and access

Our proposed framework builds on previous conceptualisations (see Table 1). Some of these have put more emphasis on characteristics of systems while others have considered the capabilities of people and populations as confounding factors [18-20,23]. However, our proposed conceptualisation is in continuous development with recent proposals suggesting that access, especially when used to reflect on the equity of a situation, should look at the resource allocation in relation to social and health needs as well as looking at geographical distribution of services linked to measures of needs and access [23,31]. Ultimately, this enables research to look at the experience of different social groups in their attempts to reach facilities [6]. It has been argued that the care an individual receives is a function of the demographic, social and economic characteristics of the family as well as characteristics of the environment in which they live [14,32]. Financial, organizational, linguistic and cultural barriers confront people wanting to use services so that, although they may have a right to health care in theory, their access may be restricted in practice [6]. Several frameworks of the determinants of utilization have been proposed and these identify with important individual, community, and health system variables [1].

### A framework based on the experiences and resistances faced by individuals

These five dimensions of accessibility of services and five abilities of potential users are embedded in the process of utilising health care and relate to causes and consequences of interacting with health providers and utilising services. They thus represent facilitators or barriers to access to health care at various stages involved in an episode of care. Barriers or facilitators can occur in a cumulative manner, from initiation of the health care seeking process to the actual benefit from available options for care. Frenk [7] already suggested the notion of resistance of health systems and utilization power of populations to explain access from a broad perspective. Resistance can be defined as the set of obstacles that arise from health resources standing in the way of seeking or obtaining care. Among these obstacles or deterrent factors are the cost of services, the location of health care sources, and certain characteristics of the ways in which the resources are organized, such as delays in obtaining appointments or in receiving care [7]. The notion of resistance could be considered as a weighted sum of obstacles. In different health systems, with varied funding mechanisms, predominant organisational models, etc. different barriers may be more prominent than in other systems [7].

Ecological obstacles arise from the location of the sources of health care, along with attendant repercussions of distances and travel time. Financial obstacles refer to the prices charged by the providers. Organizational obstacles arise from the modes of organization of health care resources. They can be divided into organizational factors at the entry point (hinders initial contact) and within the health establishment (interfere with the timely provision of care). These barriers are sequentially structured; if fundamental location and financial obstacles are overcome, then subsequently, those that arise from the organization of health care may be encountered [7].

Here we postulate the existence of a structural effect of physical and social environments on utilisation of services by individuals. This could provide researchers with ways to use variations in utilisation as markers of inequalities in access to health care. Recent studies have suggested that factors related to the characteristics of communities could be important determinants of access to health care in addition to characteristics of individuals living in urban areas and the overall availability of health care services [33,34]. Presently, there is considerable interest in geographical variations in health and the effect of context on health related problems [35]. Studies on health behaviour need to emphasise structural constraints as well as personal choices. Individuals are affected by the social, cultural, economic or physical factors acting at macro and micro levels [34,36]. Some enabling factors or barriers to access could pertain to both households and to the social environment [14,37]. The care that individuals consume is thus a function of their own demographic, social and economic characteristics as well as characteristics of the health systems and of the environment in which they live [38].

## Conclusions

Access is a concept often referred to and which has been the subject of many discussions. The objectives of this paper are to introduce a conceptualisation of access to health care describing broad dimensions and determinants that integrate demand and supply-side-factors and enables operationalisation of access to health care all along the process of obtaining care and benefiting from the services.

We have defined access as the opportunity to identify healthcare needs, to seek healthcare services, to reach, to obtain or use health care services and to actually have the need for services fulfilled. We have suggested five dimensions of accessibility (Approachability; Acceptability; Availability and accommodation; Affordability; Appropriateness) and five corresponding abilities of populations (Ability to perceive; Ability to seek; Ability to reach; Ability to pay; Ability to engage).

The proposed conceptualisation of access raises some challenges. One important challenge is the fact that measuring access is therefore not an easy task. There are of course various indicators available to measure whether or not people receive services in terms of perceived needs, if they know about available services, how they utilise services and the distance that they have to travel, on top of many measures describing the actual characteristics of services. However, a true assessment of access requires the combination of all these measures to truly judge whether the characteristics of services, providers and systems are aligned with people, households and communities capabilities.

Methodological research should enable our field to develop the measurement instruments that will better capture the complexity of access. Adding to the complexity is the fact that various sources of information can inform the varied dimensions of access, but it can be difficult to merge together to draw a complete picture of access. Mixed method analyses of consumer surveys, quality of care data, epidemiological surveys of utilisation, as well as organisational surveys may be necessary.

In addition, there is a need for more research examining the variability of access from both supply and demand-sides and looking at the influence of local health systems and patients’ characteristics. Empirical studies using the framework could also test the relevance of each dimension in different contexts and for different types of health problems and thus assess how the five provider dimensions relate to the five ability dimensions related to patients. This framework highlights the need for evaluation of strategies to improve access.

## Competing interests

The authors declare that they have no competing interests.

## Authors’ contributions

JFL designed and carried out the literature review and drafted the manuscript. MH and GR critically revised the manuscript for important intellectual content. All these authors read and approved the final manuscript.
